# The Enthusiasm of Humanity

**Published:** 1889-04-13

**Authors:** 


### The Hospital

A Weekly Institutional Journal of

**Science, Medicine, Nursing, and Philanthropy.**

*For Hospitals, Asylums, Medical Practitioners, Students, Nurses, Families, Parishes, Congregations, and the Charitable Public.*

Vol. VI. No. 133.] [April 13, 1889.

### "The Enthusiasm of Humanity."

The man who invents a telling phrase renders a decided service to the cause he has in hand. Professor Seeley, in *Ecce Homo*, made the "Enthusiasm of Humanity" popular both as a phrase and as a fact. Men and women are much better creatures than the critics will allow, or than even they will always allow themselves. Doctors live by emphasising disease, and in time they come to look upon all men as actual or potential patients. The whole world is to them more or less of a hospital, or, if not a hospital pure and simple, a hospital and a physiological laboratory combined. Similarly, the teachers of morals, finding much wickedness, which needs to be constantly denounced and, if possible, uprooted, come at last to look upon all men as downright sinners in act, or at least as grievously tainted. They quite unconsciously paint for general inspection an incorrect picture, and being public expositors who are practically unquestioned, manage to get it more or less generally approved as the correct representation of man.

It is necessary sometimes to clear one's character. "Living it down" is a method often advised, and very good in its way. But it will now and again happen that a vigorous protest and a firm refusal to be saddled with offences never committed is demanded in the interests of justice and prosperity. Similarly, mankind not seldom finds it necessary to repudiate the charges which a too metaphysical or Puritanical theology may have made against it; and to insist upon being judged, not by the artificial canons of a purely scholastic morality, but by the broad facts which commend themselves to the fairness and "common sense of most." The "Enthusiasm of Humanity" has a fine breadth about it. As with the spreading canopy of the sky, men of all colours and characters may gather themselves beneath it without any risk of inconvenient crowding. Science tells us that all human beings belong to the same natural order. That, resemblances notwithstanding, there is a sharp line of demarcation between them and the animals next highest in the scale; and that, in spite of race distinctions and peculiarities, the genus homo is practically one and indivisible. The "Enthusiasm of Humanity" is truly scientific, in that it comprehends all men.

A man of downright good sense and good feeling inevitably hates quarrelling. It is a necessity of his nature to love order and agreement. He finds in disorder and disagreement all kinds of waste, deprivation, pain, and misery. Points of contact, of amity, of mutual help, are what he looks for. Give him a classification that sunders and separates, and you make him both scientifically and morally miserable; give him a truer classification that gathers up and unites, and you satisfy his scientific instincts and his philanthropies at the same time. The "Enthusiasm of Humanity" is a uniting phrase. It offends nobody's self-love; it degrades no man unduly; it exalts no man too much. The prince and the peasant may fight under its banner, the Eastern and the Western may eat together in its shade.

The great work of "mending the lot of man" brings together vast multitudes from all the four corners of the earth. The philosopher comes to look and to take notes, and to make an ordered system. The theologian fixes his eyes on the black spots, and on those also which appear black only to him, and sets to work with his special soap and water to make them white and clean. The doctor pushes his way among the wounded, the maimed, and the sick, and, claiming them as his peculiar possession, strives with strange professional ardour to restore them to health and power of work. The philanthropist—who may be both philosopher, priest, and physician in one—regards all the woes and disabilities of man with compassion, and longs to see every human being prosperous, contented, and well. But if all this be true, human creatures are not so very detestable after all. Take away from among them a few causes of quarrel—political, ecclesiastical, social —and it is wonderful how much good they will see in each other. Fifty hounds will lie quietly together in a kennel in every kind of posture indicative of mutual confidence and affection; but if a bone be suddenly thrown in their midst, immediately pandemonium is let loose. It is so, to a large extent, with human beings. Give them a political or an ecclesiastical or other bone of contention, and they will tear each other to pieces with right goodwill. Take away their bone, and unite them in a common object about which they are all agreed, and they will be as much of one mind and heart as the hounds are on a "soft" morning with a good fox in front.

Philanthropy is a centripetal force. It disarms criticism and lulls suspicion to sleep. The good it does to those who need its help can never be calculated in terms of human speech. But though that good is so great and priceless, it is still only second to the greater reflex good which comes to philanthropists themselves and to all who in any degree temporarily or permanently join hands with them. A large company of men may be an army or a mob. Let them put forth all their strength in frequent battles against a common foe, and they become an army, enthusiastic, disciplined, invincible. Take away the common object which united them, and give them nothing to do but to criticise and dispute with each other, and you have a mob, selfish, senseless, powerless, and despicable. It is exactly so with mankind as a whole, or in separate countries. He who gives them common causes in which they can all unite is a philosopher, a statesman, a benefactor, for whom the loftiest statues and the choicest bays can never symbolise gratitude enough. The great merit of philanthropy as a common cause lies in its practicality. If a man be destitute, the fact admits of no difference of opinion. If he have a broken leg, the evidence of his calamity is patent to all men and appeals directly to every man's fellow-feeling. If a child be deaf and dumb, there is no need to discuss the possibility of the strange phenomenon or to argue that because it is contrary to common experience, therefore it cannot be real. If an octogenarian be blind, and helpless, and poor, the proof is plain, and the claim upon humanity irresistible.

In agricultural science it is well known that the sturdiest enemy to ill weeds is a thriving crop of corn or roots. The weeds cannot live where the corn is thick and strong. There is no crop so wholesome and valuable to the community as honest and intelligent philanthropy. It has been proved again and again that men of the most opposite convictions and positions in politics, religion, and other exciting questions will forget their party watchwords, doff their armour, and bury the hatchet for ever in the unity of spirit produced by the common service they are rendering to the needy and the distressed. Philanthropists will pursue their work for its own sake, and because it is purely and pre-eminently good; but they may well be encouraged also to pursue it because of its mollifying effect on the strifes and animosities of their fellows. Those, too, who have hitherto taken no part in philanthropic work, or who have looked askance at it and suspected the motives of its adherents, will do well to look at it from the point of view we have here tried to indicate. If they feel no natural aptitude and disposition for helping the sick or succouring the needy in their own hearts, they may at least consider the political and social value of that great cause which keeps strife imprisoned and sets unity and concord free. All men of experience in both family and State affairs will commend the beatitude that belongs to the peacemaker. What peace-maker, either in family or State, is so potent as goodwill carried out into good deed?

